# ‘What is my risk really?’: a qualitative exploration of preventive interventions among individuals at risk of rheumatoid arthritis

**DOI:** 10.1093/rap/rkae023

**Published:** 2024-02-29

**Authors:** Lara S Chapman, Heidi J Siddle, Stefan Serban, Kulveer Mankia, Christopher M Rooney, Zhain Mustufvi, Simon Pini, Karen Vinall-Collier

**Affiliations:** Leeds Institute of Rheumatic and Musculoskeletal Medicine, University of Leeds, Leeds, UK; Leeds Institute of Rheumatic and Musculoskeletal Medicine, University of Leeds, Leeds, UK; School of Medicine, Dentistry and Nursing, University of Glasgow, Glasgow, UK; Leeds Institute of Rheumatic and Musculoskeletal Medicine, University of Leeds, Leeds, UK; NIHR Leeds Biomedical Research Centre, Leeds, UK; Leeds Institute of Medical Research, University of Leeds, Leeds, UK; School of Dentistry, University of Leeds, Leeds, UK; Leeds Institute of Health Sciences, University of Leeds, Leeds, UK; School of Dentistry, University of Leeds, Leeds, UK

**Keywords:** rheumatoid arthritis, prevention, qualitative, interviews, behaviour

## Abstract

**Objectives:**

Intervention in the pre-arthritis phase of RA could prevent or delay the onset of disease. The primary aim of this study was to explore perspectives of being at risk and potential preventive interventions among individuals at risk of developing RA and to identify factors influencing their engagement with prevention. A secondary aim, established during the analytical process, was to understand and compare different approaches to health-related behaviours related to prevention of RA.

**Methods:**

Anti-CCP-positive (CCP^+^) at-risk individuals with musculoskeletal symptoms but no synovitis participated in semi-structured interviews. Data were analysed using reflexive thematic analysis, followed by a secondary ideal-type analysis.

**Results:**

Nineteen CCP^+^ at-risk individuals (10 women; age range 35–70 years) participated. Three overarching themes were identified: being CCP^+^ at risk; aiming to prevent RA; and influencers of engagement. Participants described distress related to symptoms and uncertainty about disease progression. Many participants had concerns about medication side effects. In contrast, most participants expressed willingness to make lifestyle changes with the aim of preventing RA. Engagement with preventive measures was influenced by symptom severity, personal risk level, co-morbidities, experiences of taking other medications/supplements, knowledge of RA, risk factors and medications, and perceived effort. Three types of participants were identified from the data: proactive preventers, change considerers and fearful avoiders. Overall orientation to health behaviours also impacted the attitude towards preventing RA.

**Conclusion:**

Findings could inform recruitment and retention in RA prevention research and promote uptake of preventive interventions in clinical practice.

Key messages

- This study could inform RA prevention trial design, including eligibility criteria, recruitment and retention strategies.
- Engagement with prevention is influenced by knowledge about risk and experiences of taking other medications.
- An at-risk individual’s overall orientation to health behaviours can also impact on attitude towards prevention.

## Introduction

A preclinical phase of RA, during which at-risk individuals develop autoantibodies and/or symptoms before progressing to clinical arthritis and classifiable RA, is well established [1]. Evidence suggests that intervention in the pre-arthritis phase could reduce the likelihood of RA developing [2], delay onset of RA [3] or reduce the severity of the condition should it develop [4].

An increasing number of clinical trials investigating therapeutic approaches in at-risk individuals, with the aim of preventing RA, are underway [5, 6]. In addition to modulating the immune response with disease-modifying drugs, prevention can also target other risk factors. For example, smoking, elevated BMI and dyslipidaemia have been identified as risk factors for the development of RA in at-risk individuals [7]. Research is also focusing on inflammation and associated autoantibodies as risk factors for RA. For example, there is increasing interest in how the risk of developing RA could be reduced by improving oral health, specifically by reducing periodontal inflammation [8, 9]. Additionally, anti-CCP-positive (CCP^+^) at-risk individuals appear to have a distinct gut microbiome compared with healthy controls [8], suggesting a role for microbiome-based therapeutics in preventing RA [9]. Addressing RA risk factors also has the potential to provide broader health benefits [10–12].

It is important that potential treatments are acceptable to at-risk individuals, particularly given that RA prevention trials to date have demonstrated recruitment challenges [13–15]. At-risk individuals have expressed concerns regarding taking preventive medication owing to potential side effects, whereas lifestyle changes, such as healthy eating, increased exercise and smoking cessation, are perceived to be more acceptable [16]. A recent qualitative study identified that periodontal treatment and oral health maintenance are also potentially acceptable preventive measures [17]. There is a pressing need for further understanding of how at-risk individuals weigh up the risk of developing RA *vs* the benefits of preventive interventions [18]. Furthermore, determining preferences for lifestyle approaches to reduce the risk of RA development in at-risk groups has been identified as an important topic for further study [19, 20].

The primary aim of this study, established *a priori*, was to explore perspectives of being at risk and potential preventative interventions. A secondary aim was established during familiarization with the data, whereby an ideal-type analysis was conducted to identify and describe different patterns of thinking among these participants and to understand and compare different approaches to health-related behaviours.

## Methods

This was a qualitative interview study using a phenomenological approach. It is reported in line with the Consolidated Criteria for Reporting Qualitative Studies (COREQ) framework [21] (Supplementary Data S1, available at *Rheumatology Advances in Practice* online). The present study uses a dataset gathered in a previous project [20].

### Patient and public involvement

Patient and public involvement (PPI) partners from local dental and rheumatology PPI groups were involved in shaping the research question and in developing the interview topic guide and participant information sheet for the study. PPI contributors also informed our approach to data collection; as result of their input, all participants were offered the choice between a video or telephone interview to enhance inclusivity.

### Participants

A purposive sample of individuals at risk of developing RA was recruited from the Leeds CCP cohort. This national research cohort recruits and follows individuals who present with new non-specific musculoskeletal symptoms but no synovitis and test positive for anti-CCP antibodies. Individuals aged ≥18 years who were able to give informed consent and who were able to speak and understand English were eligible to participate. Potential participants were approached by a member of the CCP cohort research support team, by telephone, and invited to take part in a single interview focusing on the acceptability of dental care and other measures aiming prevent RA. This study complies with the Declaration of Helsinki. Ethical approval was granted by Cambridge East REC (ref. 20/EE/0230). Electronic informed consent was obtained from all participants.

### Data collection

Individual semi-structured interviews were conducted via video or telephone by one of two researchers (K.V.-C., a psychologist and senior qualitative researcher in dental public health, and H.J.S., a podiatrist with expertise in pre-RA research; both PhD and female) between March and June 2022, using a topic guide (Supplementary Data S2, available at *Rheumatology Advances in Practice* online). The motivations of both researchers to improve engagement with preventive measures and their previous experiences of prevention in research and clinical practice influenced the data collection process, e.g. in building rapport with participants during the interviews. One other researcher (L.S.C.) observed two interviews. All researchers were previously unknown to the participants. The topic guide was developed by the multidisciplinary clinical research team (with input from rheumatology, dental and microbiology specialisms) and piloted with PPI contributors. Questions were open ended and covered symptoms, risk status, oral health, diet, smoking cessation, medication used to treat RA, probiotics and antibiotics.

All participants received information on the purpose of the study, and the interviewer’s background and personal motivation. Participants were given the opportunity to ask questions before participating. All participants provided electronic informed consent. The audio of all interviews was digitally recorded, transcribed verbatim and supplemented with field notes. The duration of the interviews ranged from 23 to 45 min. The final sample size was based on achieving adequate diversity of the sample and depth of data generated.

### Analysis

Transcripts were analysed using reflexive thematic analysis to identify patterns in meaning across the dataset to identify themes [22]. Interviews were uploaded into NVivo v.12 (QSR International, 2018) and initially coded by one researcher (L.S.C.), who read and re-read the transcripts, generated initial codes and collated similar codes. The researcher made reflective notes relating to each participant’s perspectives towards prevention when initially reading each transcript, and repeatedly returned to the transcripts and the reflective notes throughout the entire analysis process to ensure that it was firmly grounded in the data [23]. One other researcher (K.V.-C.) independently coded 10% of the transcripts. Regular coding discussions were held with all other team members, and discrepancies were settled by group consensus. Members of the research team working in a clinical practice capacity with at-risk individuals (H.J.S. and K.M.) reflected and drew upon their personal experiences of encouraging engagement with prevention during the analysis process.

During the thematic analysis, specific types were identified, reflecting different responses to being at risk of RA and different approaches to health behaviours aiming to prevent onset. These types appeared to underpin the data and influence the way in which participants related to the research topic. Therefore, a secondary ideal-type analysis was conducted to identify and describe different patterns of thinking among these participants and to understand and compare different approaches to health-related behaviours [24]. Emerging ideas and notes made by the researchers during the thematic analysis led to the construction more latent codes that formed the basis of this ideal-type analysis, which sought to focus on ‘deeper, more implicit or conceptual level of meaning’ [23].

The ideal-type analysis involved systematically comparing cases within the qualitative dataset to form groupings of similar cases. The analysis followed stages recommended by Stapley *et al.* [24]. Two researchers (H.J.S. and K.V.-C.) independently read the transcripts, wrote case reconstructions, constructed ideal types, then identified optimal cases and formed ideal-type descriptions through group discussion. To ensure that descriptions of the ideal types were appropriately grounded in the data, a third researcher (L.S.C.) independently grouped cases into the ideal types. Further discussion among the three researchers led to regrouping and rewording of the ideal-type descriptions.

## Results

Nineteen CCP^+^ at-risk individuals (10 women; age range 35–70 years) participated (Table 1). An additional three participants were approached but declined participation owing to ill health or development of inflammatory arthritis.

**Table 1.** At-risk participant characteristics

Participant no.	**Gender**	Age at interview (years)	Smoking (ever, previous or current/never)	**Musculoskeletal symptoms** ^a^ **(yes/no)**	Relative with RA (yes/no)
1	Female	60	Never	Yes	No
2	Male	41	Never	Yes	No
3	Female	55	Never	Yes	No
4	Female	56	Never	Yes	No
5	Female	50	Ever, previous	Yes	Yes (grandmother)
6	Male	42	Never	Yes	Yes (mother)
7	Male	53	Never	Yes	No
8	Male	70	Ever, current	No	No
9	Female	35	Ever, current	Yes	No
10	Female	62	Never	Yes	No
11	Female	55	Never	Yes	No
12	Male	61	Never	Yes	Unconfirmed (grandmother)
13	Male	54	Ever, current	Yes	Yes (grandfather)
14	Male	38	Ever, current	Yes	Yes (mother)
15	Female	40	Never	Yes	No
16	Male	60	Ever, current	Yes	No
17	Female	56	Ever, previous	Yes	No
18	Male	56	Never	Yes	Yes (father)
19	Female	52	Ever, previous	Yes	No

a Musculoskeletal symptoms within 9 months preceding the interview. All participants had musculoskeletal symptoms when first included in the CCP study.

### Thematic analysis

**Figure 1.**
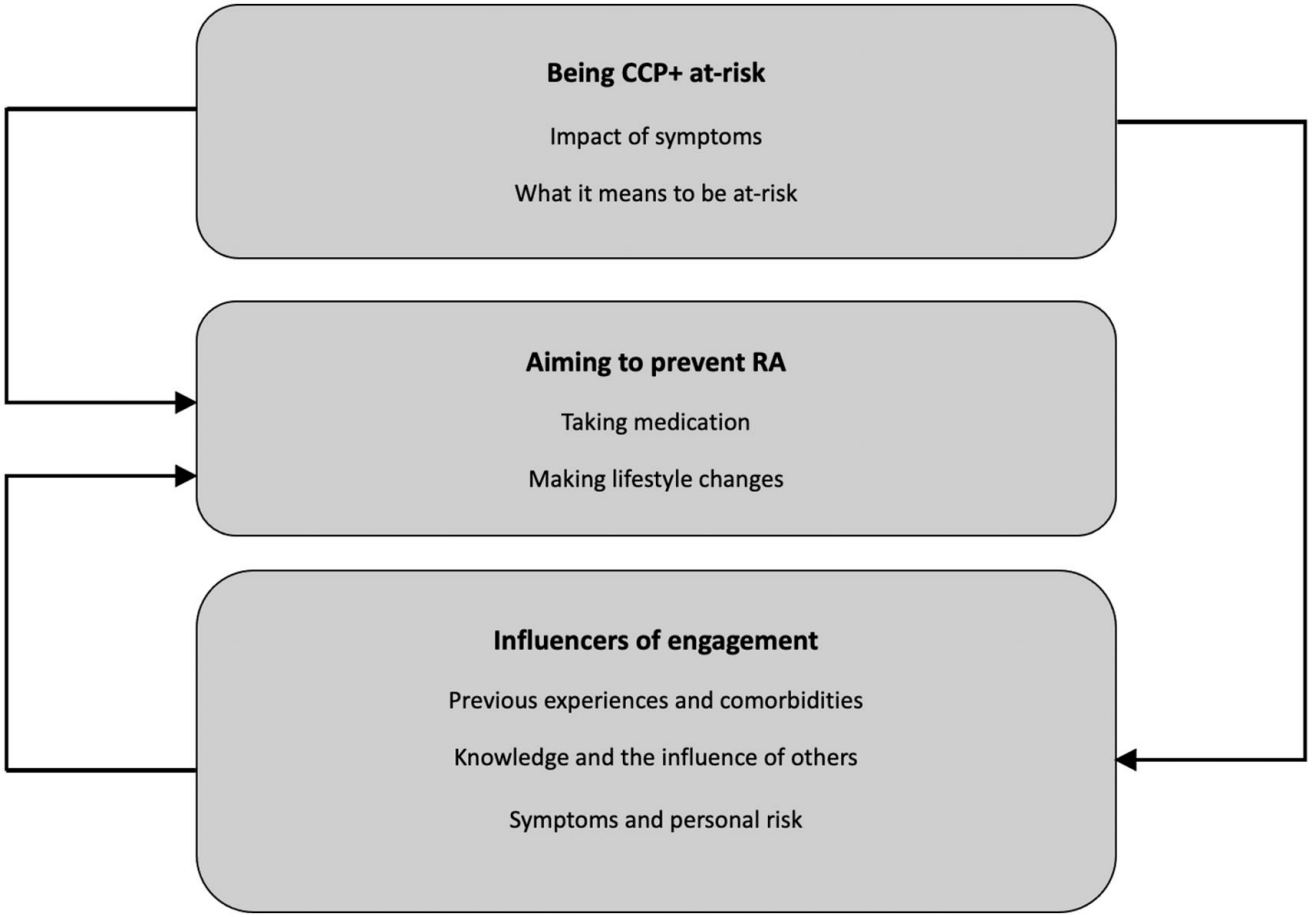
Thematic map

Three overarching themes (seven subthemes) were identified from the thematic analysis; a conceptual thematic map is presented in Fig. 1.

#### Theme 1: being CCP^+^ at risk

Within theme 1, two subthemes were identified relating to experiences of being at risk of developing RA, encompassing the impact of RA symptoms and knowledge about what it meant to participants to be ‘at risk’. Verbatim quotations supporting theme 1 are presented in Table 2.

**Table 2.** Participant verbatim quotes for theme 1 (being CCP^+^ at-risk)

Quote no.	Verbatim quote
Q1	They gave me a laptop to work from home if I needed to, and things like that, but it were just not being able to do the job I were paid to do … I didn’t want to sit at home in the office, you know, I just wanted to do what I were paid to do, so I’ve had to cut short my career. – Participant 11
Q2	It just makes you feel a bit sad as well, let’s be honest, you know, it gets you down, cause obviously with being quite young as well, it’s very, I’m very much I’m out there and active and I have to pull that back a bit as well, which got to me quite a bit. – Participant 9
Q3	Well, I would say if it was the Grand National, I’d be odds on favourite to win arthritis. The arthritis stakes. – Participant 7
Q4	I think it’s reasonably high levels though I don’t know what the norm is and what high, medium and low levels are. – Participant 1

*Subtheme 1.1. Impact of symptoms.* Participants described the symptoms synonymous with RA that led to involvement in the CCP cohort study and the physical and emotional impact of these symptoms. Joint pain led to limitation or modification of activities, being unable to work, and relying on family members for personal care (Table 2, quote 1). In contrast, one participant noted that despite having occasional symptoms, being CCP^+^ at risk had not impacted upon his life. Other participants described ‘getting on with it’ and compared themselves to others with conditions they perceived to be worse:

It’s not life defining at the minute, it’s painful when it happens, when I get a bout of it, it’s painful, but there’s a lot worse people off than me, so I suffer. Begrudgingly grin and bear it. – Participant 16

Ongoing symptoms and the uncertainty of whether RA would develop in the future caused emotional distress among participants, including fear, worry and sadness (Table 2, quote 2). In addition to experiencing symptoms, participants’ perceptions of developing RA were informed by knowing others with the condition, e.g. relatives, and by reading about it. Participants perceived that RA would be painful, lead to a reliance on others, and could reduce mobility and stop them from doing their daily activities.

*Subtheme 1.2. What it means to be at risk.* All participants had some understanding what it meant to be ‘at risk’; many referred to a marker in their blood. Some had further knowledge about the level of their risk but varied in their understanding of what this meant for them personally (Table 2, quotes 3 and 4). One participant perceived that knowing his risk level would help him to plan ahead. Likewise, another participant wanted to know how severe the RA would be if it developed, and what impact it would have on his life:

It can progress at absolutely any time, it could be tomorrow that it could progress, or it could be 10 years, or it could be 5 … I don’t know. Is it … volatile, ’cause you can’t predict it, and if it does hit, when it hits, it’s gonna be life changing? That to me makes it a much riskier condition to sort of ignore. – Participant 2

#### Theme 2: aiming to prevent RA

Within theme 2, participants’ perspectives on medication and lifestyle changes aiming to prevent RA from developing were identified in two subthemes relating to the type of preventive intervention. Verbatim quotations supporting theme 2 are presented in Table 3.

**Table 3.** Participant verbatim quotes for theme 2 (aiming to prevent RA)

Quote no.	Verbatim quote
Q5	I wouldn’t call it as risky. You know, it’s like taking paracetamol and everything has a side effect. I am aware that there could be side effects and it’s more invasive than having a yogurt or antibiotic … but if it could prevent me not doing my day-to-day activities, then why not? But that’s the mindset I’ve got really … I’m open-minded in that sense. – Participant 15
Q6	They told me to exercise and be careful of my weight, and I’ve done that. And also, I take vitamin D as well. – Participant 10
Q7	I do a lot of exercise, cause I’ve got a dog and I walk him a lot. That’s kind of why I got him really … I don’t do necessarily heavily physical sports and contact sports or anything like that, but at least with walking, get a lot of mileage in that way. – Participant 14
Q8	They actually did mention teeth, and I think I upped that at the time, going to the hygienist a bit more because I’ve had a bout of gum disease and stuff related to arthritis. – Participant 10

*Subtheme 2.1. Taking medication.* Most participants had concerns about taking medication that is used to treat RA with the aim of prevention owing to perceived side effects:

I don’t particularly like taking drugs anyway, you know. If there’s a way to combat it without taking a drug, that seems a better idea than actually, oh, give you some tablets, take them and it might do something, and it might not, you know. I’d rather see if it could clear without lab tablets, without medication. – Participant 5

In contrast, one participant did not have concerns, despite acknowledging potential side effects (Table 3, quote 5). Some participants were more willing to consider taking preventive antibiotics than RA medications, although this was dependent upon the evidence supporting their use, length of the course of treatment and side effects. Most participants were willing to take a probiotic to prevent or delay the onset of RA; side effects were a concern to only one participant. Participants perceived probiotics to be low risk, non-invasive and easier than taking a drug.

*Subtheme 2.2. Making lifestyle changes.* Participants perceived that good general health, including exercising, eating healthily and managing their weight and stress levels, would help to prevent the development of RA, and most participants had engaged with some of these lifestyle changes as a result of being at risk (Table 3, quotes 6 and 7). Among participants who were current smokers, all expressed willingness to attempt to stop or reduce smoking with the aim of preventing RA:

I suppose yeah, I’d be prepared to stop if you thought it’d help … I don’t think I could stop altogether, but, you know, cut down the amount I smoke. – Participant 13

Additionally, most participants felt that oral health treatment and maintenance would be an acceptable measure aiming to prevent RA (Table 3, quote 8).

#### Theme 3: influencers of engagement

Factors influencing participants’ perceived or actual engagement with measures aiming to prevent RA were identified in theme 3, within three subthemes (previous experiences, knowledge and the influence of others, and symptoms and personal risk). Verbatim quotations supporting theme 3 are presented in Table 4.

**Table 4.** Participant verbatim quotes for theme 3 (influencers of engagement)

Quote no.	Verbatim quote
Q9	I had pylori a few years ago and I had to take antibiotics. That set me back easily 6 months … I felt bloated all the time, I had heartburn for a long time, so until I got my gut back from those antibiotics, it took me ages, so now when someone says anything about antibiotics I just get like not really. I try to avoid them. – Participant 6
Q10	That’s fine. I took a whole 4-week course of antibiotics for high pylori, which I didn’t even know I had. – Participant 15
Q11	With being diagnosed recently as type 2 … your eating habits sort of have to change slightly. So I’ve cut out processed meat and bread, trying to be a little bit healthy, trying to get a little bit more fish into the diet. – Participant 16
Q12	Some people probably try to be more healthy and more things, but I have a lot of stress and anxiety as well, so I stress myself out more. You know, like not enjoying certain things. – Participant 14
Q13	I already take, it’s a bit hit and miss, but I have, I do take vitamin D tablets in the winter, and I’m also taking some vitamin tablets for my eyes. – Participant 2
Q14	I mean, you get older, and I don’t feel good after sugar anyway, so … it’s lost a fair bit of its joy, kind of thing. So, yeah, it’s been a long time since I’ve done much eating between meals and skipping breakfast and all that sort of stuff. – Participant 19
Q15	It’s at work really, ’cause you get bored, so you go out for a cig … but I’m trying to use the vape. – Participant 13
Q16	If somebody told me, well you’ve got to eat, I don’t know, oily fish five times a week. That sounds excessive. But, like, you know, if there’s some specific guidance that they can give, you know, if you’re gonna do anything, do this one thing, then absolutely, yeah, I’d definitely take that on board and I’d start doing it. – Participant 2
Q17	I changed a bit my diet. That’s gonna be quite a long story, but my mum also has gluten intolerance, which was diagnosed at the same time as the rheumatoid arthritis … definitely with gluten from the diet, she’s got a lot better. She managed to manage the progression of the illness. – Participant 6
Q18	Me husband had a heart attack, and he smoked. – Participant 5
Q19	[Tablets are for] when it gets to the extreme, but if there are other alternatives I would probably prefer the other alternatives. – Participant 4
Q20	If your marker was, I don’t know, out of ten, you’re up at five. I’d be like, oh really … but if I was like a nine or an eight, I’d be like, well yeah, get me on it quick, shut up. – Participant 15

*Subtheme 3.1. Previous experiences.* Participants’ perceived engagement with preventive measures was influenced by their previous experiences of medication. Those who had experienced negative side effects from drugs in the past were less willing to take preventive RA medication and preventive antibiotics (Table 4, quote 9). In contrast, participants who had taken certain drugs previously with no negative experiences were more willing to take medication with the aim of preventing RA (Table 4, quote 10). Likewise, participants who had experienced a medication working for them were more willing to take it than those who had experienced a medication not working. Some participants who were taking preventive medication for conditions other than RA were more willing to consider taking medication aiming to prevent RA:

I take statins so they’re going along the same lines I presume in one way, trying to prevent something. So possibly, yeah. – Participant 12

Conversely, some participants who were already taking medications were apprehensive about taking more:

If I take up to four Nurofen, two in the morning and two on a night, then I can avoid a problem, but I don’t wanna be taking a load of tablets, because I’m already taking tablets, you know, I’ll rattle when I walk. – Participant 7

Some participants had made lifestyle changes owing to other conditions, including diabetes, kidney stones, heart disease and high cholesterol (Table 4, quote 11). However, co-morbidities could also negatively impact upon engagement with RA prevention measures, particularly mental health issues and when other physical conditions took priority (Table 4, quote 12). Some participants recognized the ease of making certain changes with the aim of preventing RA, such as taking probiotics, eating healthily and maintaining oral health, based on how these aligned with their current lifestyle. For example, preventive probiotics were likened to other vitamins or supplements. Other participants reported having adopted a healthy diet independently of their RA risk (Table 4, quotes 13 and 14). In comparison, some lifestyle changes were perceived to require greater effort. Smoking cessation was perceived to be a particularly high-effort action and was reported to be influenced by having a busy lifestyle, work and stress (Table 4, quote 15).

*Subtheme 3.2. Knowledge and the influence of others.* Participants’ perceived willingness to engage with measures aiming to prevent RA was also influenced by their knowledge of risk factors. Most participants had been unaware of any potential link between gut health or oral health and the development of RA; once given this information, some expressed willingness to engage. Knowledge of the preventive measure itself was also influential:

As long as it’s not the type of antibiotic that you become resistant to. But there are different types aren’t there, so yeah, I would accept that, yeah. – Participant 1

But you only take antibiotics if you’ve got an infection, don’t you? – Participant 10

Other participants highlighted the need for more advice around preventive measures, such as diet (Table 4, quote 16). Knowledge of and engagement with measures aiming to prevent RA was also influenced by relatives, friends and acquaintances with RA (Table 4, quote 17). For one participant, the influence of another condition on her husband led her to stop smoking (Table 4, quote 18).

*Subtheme 3.3. Symptoms and personal risk.* Participants’ perceived engagement with preventive measures was influenced by the presence and impact of symptoms (Table 4, quote 19). Some participants perceived that having symptoms was enough to engage, regardless of their personal risk. Perceived willingness to engage with measures aiming to prevent RA could also depend on personal risk level, with a higher personal risk being linked to increased engagement (Table 4, quote 20). Comparatively, another participant perceived that having a lower CCP level was a motivating factor to make changes aiming to prevent RA:

I think if I knew it was really high, I’d probably be more worried, but I felt that the level I had wasn’t really, really high and that I could do something about it. – Participant 10

One participant who smoked felt that a better understanding of her personal risk level wouldn’t necessarily lead to smoking cessation. Other participants highlighted the need to consider their personal risk of developing RA against their symptoms when considering whether or not to take preventive medication.

### Ideal-type analysis

**Figure 2.**
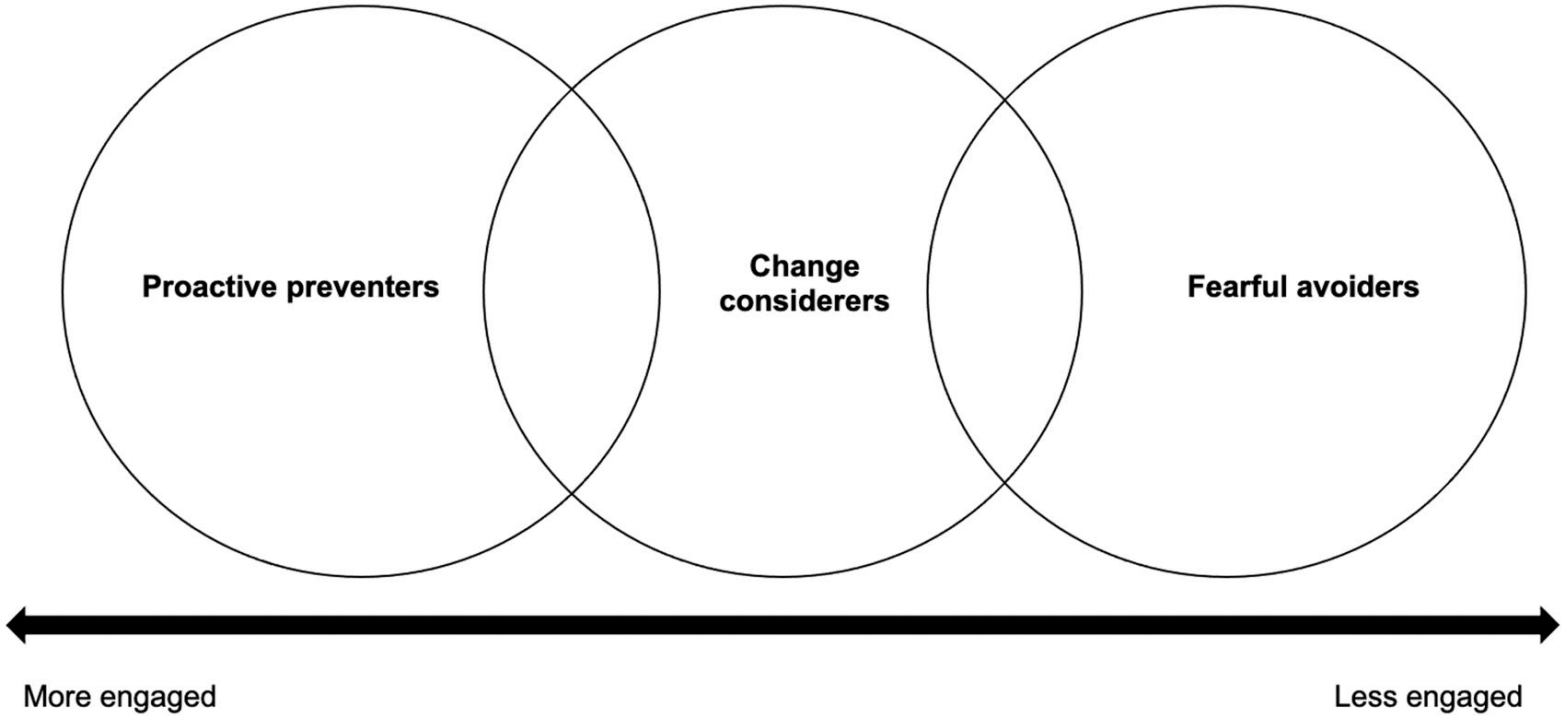
Typology of individuals at risk of RA

Three ‘types’ of participants were identified from the data: proactive preventers, change considerers and fearful avoiders (Fig. 2). However, some overlap between types was observed, and participants demonstrated elements of different types depending on the context. The types broadly represent attitudes towards being at risk and prevention in general, particularly with regard to making lifestyle changes, although willingness to take preventive RA medication and preventive antibiotics varied across the three types and was highly dependent upon the factors identified in theme 3.

#### Type 1: proactive preventers

Participants in this group (*n* = 8) tended to demonstrate a good understanding ‘that there’s elevated CCP in your blood, which means that you have higher risk of developing arthritis’ (Participant 10). These participants were health conscious; they sought information relating to reducing their risk of developing RA and acted on this knowledge. Participants were self-motivated to make lifestyle changes; in some cases, they reported having healthy lifestyles regardless, but being at risk and seeking further knowledge often affirmed existing lifestyle choices and led to further positive changes, because ‘risk makes you pay attention’ (Participant 10) and ‘the whole point is to try and prevent it before it becomes fully blown arthritis and try and nip it before it becomes debilitating’ (Participant 15). Participants in this group were generally willing to consider the concept of prevention aiming to prevent RA, suggesting ‘yeh, anything to make it better’ (Participant 8), ‘anything that can help me, I’m willing to do’ (Participant 17), ‘I’ll do whatever is required to reduce the risk’ (Participant 6) and ‘I’d rather start [medication] the earlier the better’ (Participant 9).

#### Type 2: change considerers

Participants in this group (*n* = 9) had less knowledge of what being at risk meant, suggesting it was ‘some sort of marker in me blood—not quite sure what it shows, but it’s something to do with I may or may not get rheumatoid arthritis’ (Participant 5), and demonstrating misconceptions about the disease process: ‘at the moment I’ve got osteo, so touch wood it doesn’t progress to rheumatoid’ (Participant 18). These participants considered making changes to reduce their risk of developing RA and some overlap between change considerers and proactive preventers was evident, but implementing changes often depended upon the effort involved and could be affected by other priorities. Some participants would rather not worry about developing RA yet, as ‘you can’t worry about everything, ’cause if you worry about everything, you’d never do anything, would you, you’d just stay in a locked room all day … I’ll read all about it when I get it’ (Participant 18), and instead were more likely to ‘wait and see’, hoping they ‘get lucky’ and ‘hoping for the best’ (Participant 1). These participants ultimately dealt with health issues, including worsening symptoms, as they happened and compared themselves to others who they perceived to be worse off. Additionally, participants in this group tended to rely on direct medical advice to make changes, rather than seeking out knowledge themselves, with the onus on the health professional: ‘I would [make dietary changes] if a health-care professional suggested it’ (Participant 12); ‘I mean if you prescribe, give me summat, then yeah’ (Participant 16); and ‘you can sign me up [to an RA prevention study] if you want’ (Participant 13).

#### Type 3: fearful avoiders

A minority of participants (*n* = 2) exemplified this type, although elements of this type were also present in the accounts of other participants. Participants felt ‘a bit scared of what the implications [of being at risk] might be’ (Participant 19). However, they had limited knowledge or engagement relating to being at risk, revealing they had not explored condition in depth, ‘just seen it as … it could go either way really’ (Participant 14), and did not want to ‘look into it until … well not even at all actually’ (Participant 19). Likewise, engagement with health-related behaviour change was limited: ‘I’m probably guilty of not living the healthy lifestyle’ (Participant 14). These participants also revealed ongoing unhealthy habits, such as smoking and poor diet, but did acknowledge some efforts to be healthier, e.g. physical activity.

The most apparent overlap of types was between fearful avoiders and change considerers; some change considerers implied avoidance, especially in relationship to RA and what it means to be at risk, whilst fearful avoiders were willing to consider making changes to prevent RA, particularly those relating to their lifestyle.

## Discussion

Our findings provide new insight into perceived engagement with a range of measures aiming to prevent RA among individuals at risk of developing the condition. Additionally, we have identified three types of approaches to health-related behaviours that individuals at risk of RA might take. Approaching RA prevention presents an ongoing challenge in rheumatology. Although early initiation of immunotherapies in at-risk individuals is encouraging, previous studies have shown that many at-risk individuals have concerns about pharmacological side effects [13, 16, 19]. In congruence, our findings suggest that side effects associated with the medication used to treat RA could cause concern for at-risk individuals, regardless of how proactive they might be in other ways. Also in line with previous studies, perceived engagement with preventive measures was influenced by symptom severity and personal risk level, with more severe symptoms and higher personal risk leading to increased willingness [16]. Our findings suggest that experiences of taking other medications or supplements, co-morbidities, knowledge of RA and its risk factors, and perceived effort relating to the intervention in question might also influence engagement.

Our study is the first to explore the perceptions of at-risk individuals of measures aiming to address the gut microbial dysbiosis implicated in development of RA. Similar to perceptions of the medications used to treat RA, antibiotics were perceived to have negative side effects. However, personal experiences of taking these previously also influenced perceptions. In line with previous research [25], we identified some misconceptions around antibiotics among participants. In comparison, most participants indicated that taking probiotics with the aim of preventing RA would be acceptable.

We acknowledge some limitations to our study. Firstly, despite the valuable insights offered through the secondary analysis, our sample size was smaller than recommended for ideal-type analysis [24]. Notwithstanding, this is the first study to identify differences in orientations to health behaviours in this population and provides a grounding for further qualitative research in this area. Secondly, the ideal-type analysis was not the primary focus of this study, and although we aimed to understand different attitudes towards prevention of RA in the interviews, we did not explicitly explore typology during data collection, and our data might not represent all types. Furthermore, we acknowledge that our topic guide questions were weighted towards preventive measures related to oral health. However, responses relating to other preventive measures were explored in detail and generated rich information. Although PPI contributors were involved in the design of our topic guide, these were not individuals who were at risk of developing RA; involvement of at-risk individuals in the design and analysis might have enhanced our study. Finally, participants in our study were recruited from an existing research cohort, which potentially facilitates recruitment of more proactive preventers and fewer fearful avoiders by its very nature; our findings therefore might not reflect the full range of perceptions among individuals at risk of developing RA, and further work is needed to understand the attitudes of at-risk individuals who do not typically get involved in research, e.g. those who declined participation or dropped out of the Leeds CCP cohort study.

When approaching RA prevention, individuals at risk of RA might need differing levels of information and support, both in practice and in relationship to recruitment and retention in clinical trials. Our findings indicate that some at-risk individuals take an active interest in their risk status and are highly engaged in prevention; these individuals could be given as much information and advice as early as possible, in addition to feedback on changes they have already made. Other at-risk individuals will generally follow advice from health professionals but are not necessarily interested in details surrounding the development of RA and potential prevention. These individuals potentially need reiteration of information relating to what it means to be at risk, advice about specific aspects of prevention depending on their personalized risk factors and increasing information about the disease itself at later time points, e.g. if their symptoms become more severe. Finally, some at-risk individuals are much less likely to engage with information about RA and preventive measures but might express willingness to make some small changes to improve their overall health. Assessing readiness to change, building motivation and setting SMART (specific, measurable, achievable, relevant and timely) goals are key areas to focus on [26, 27]. Overall, our findings suggest that recruitment to clinical trials could be improved by focusing on approaching specific types of at-risk individuals, depending on the trial. For example, at-risk individuals with symptoms, high perceived personal risk level, proactive personality and positive experiences of taking medication could be approached for drug trials in this area.

## Conclusion

Having more severe symptoms, a higher personal risk level, knowledge about risk factors and positive experiences of taking other medications might increase engagement with preventive interventions among CCP^+^ at-risk individuals, but an individual’s overall orientation to health behaviours also impacts on their attitude towards preventing RA. These findings could inform recruitment and retention in RA prevention research and promote uptake of preventive interventions in clinical practice. Understanding specific barriers and facilitators to the different ideal types of patients will be beneficial to increase engagement with preventive interventions in individuals at risk and will help clinicians to recruit and maintain patient participation in preventive intervention studies.

## Supplementary Material

rkae023_Supplementary_Data

## Data Availability

The data that support the findings of this study are not publicly available as they contain information that could compromise the privacy of research participants.
