# Supplementary material for: ‘What is my risk really?’: a qualitative exploration of preventive interventions among individuals at risk of rheumatoid arthritis
Source: Rheumatol Adv Pract. 2024 Feb 29;8(2):rkae023. doi: 10.1093/rap/rkae023 (PMC10939459; doi:10.1093/rap/rkae023)
Supplement: rkae023_Supplementary_Data [file rkae023_supplementary_data.docx]

**Supplementary Data S1. Completed Consolidated criteria for reporting qualitative studies (COREQ): 32-item checklist**

Developed from:

Tong A, Sainsbury P, Craig J. Consolidated criteria for reporting qualitative research (COREQ): a 32-item checklist for interviews and focus groups. *International Journal for Quality in Health Care*. 2007. Volume 19, Number 6: pp. 349 – 357

| **No. Item** | **Guide questions/description** | **Reported on Page No.** |
| --- | --- | --- |
| **Domain 1: Research team and reﬂexivity** |  |  |
| *Personal Characteristics* |  |  |
| 1. Interviewer/facilitator | Which author/s conducted the interview or focus group? | Page 5 |
| 2. Credentials | What were the researcher’s credentials? E.g. PhD, MD | Page 5 |
| 3. Occupation | What was their occupation at the time of the study? | Page 5 |
| 4. Gender | Was the researcher male or female? | Page 5 |
| 5. Experience and training | What experience or training did the researcher have? | Page 5 |
| *Relationship with participants* |  |  |
| 6. Relationship established | Was a relationship established prior to study commencement? | Page 5 |
| 7. Participant knowledge of the interviewer | What did the participants know about the researcher? e.g. personal goals, reasons for doing the research | Page 5 |
| 8. Interviewer characteristics | What characteristics were reported about the interviewer/facilitator? e.g. Bias, assumptions, reasons and interests in the research topic | Page 5 |

| **Domain 2: study design** |  |  |
| --- | --- | --- |
| *Theoretical framework* |  |  |
| 9. Methodological orientation and Theory | What methodological orientation was stated to underpin the study? e.g. grounded theory, discourse analysis, ethnography, phenomenology, content analysis | Page 4 |
| *Participant selection* |  |  |
| 10. Sampling | How were participants selected? e.g. purposive, convenience, consecutive, snowball | Page 4 |
| 11. Method of approach | How were participants approached? e.g. face-to-face, telephone, mail, email | Page 4 |
| 12. Sample size | How many participants were in the study? | Page 6 |
| 13. Non-participation | How many people refused to participate or dropped out? Reasons? | Page 6 |
| *Setting* |  |  |
| 14. Setting of data collection | Where was the data collected? e.g. home, clinic, workplace | Page 5 |
| 15. Presence of non-participants | Was anyone else present besides the participants and researchers? | Page 5 |
| 16. Description of sample | What are the important characteristics of the sample? e.g. demographic data, date | Page 6-7 (Table 1) |
| *Data collection* |  |  |
| 17. Interview guide | Were questions, prompts, guides provided by the authors? Was it pilot tested? | Page 5 |
| 18. Repeat interviews | Were repeat interviews carried out? If yes, how many? | N/A – single interview (Page 5) |
| 19. Audio/visual recording | Did the research use audio or visual recording to collect the data? | Page 5 |
| 20. Field notes | Were ﬁeld notes made during and/or after the interview or focus group? | Page 5 |
| 21. Duration | What was the duration of the inter views or focus group? | Page 5 |
| 22. Data saturation | Was data saturation discussed? | Page 5 |
| 23. Transcripts returned | Were transcripts returned to participants for comment and/or correction? | N/A – transcripts were not returned. |
| **Domain 3: analysis and ﬁndings** |  |  |
| *Data analysis* |  |  |
| 24. Number of data coders | How many data coders coded the data? | Page 5 |
| 25. Description of the coding tree | Did authors provide a description of the coding tree? | N/A – not provided. |
| 26. Derivation of themes | Were themes identiﬁed in advance or derived from the data? | Page 5 |
| 27. Software | What software, if applicable, was used to manage the data? | Page 5 |
| 28. Participant checking | Did participants provide feedback on the ﬁndings? | N/A – no. |
| *Reporting* |  |  |
| 29. Quotations presented | Were participant quotations presented to illustrate the themes/ﬁndings? Was each quotation identiﬁed? e.g. participant number | Pages 8-17 |
| 30. Data and ﬁndings consistent | Was there consistency between the data presented and the ﬁndings? | Pages 8-17 |
| 31. Clarity of major themes | Were major themes clearly presented in the ﬁndings? | Pages 8-17 |
| 32. Clarity of minor themes | Is there a description of diverse cases or discussion of minor themes? | Page 8-17 |

**Supplementary Data S2. Topic guide (at-risk participants)**

**RA/Systemic health**

- - When were you first told about your risk of arthritis? CCP+ test?
    - - How did you receive this news?
      - Do you understand what this risk means?
  - What do you understand by someone having RA?
    - - Do you have family members with RA or any experience of RA?
      - Do you have any symptoms? If so what? E.g. joint pain
      - What is the impact on your life/if any? E.g. fatigue
  - Have your health priorities changed since you found out you were at risk of developing RA?
    - Could you describe your top 5 health priorities since CCP+ test?
      - What about your teeth and gums?
      - How important is this?

What do you understand around the link between gum disease and RA? Have you ever heard about a link before?

**Barriers and facilitators for keeping your mouth healthy?**

- - - Do you feel you have healthy teeth and gums?
      - If not- why?
      - What makes you think that?
      - Is there anything you would like to change/would you like to improve your teeth and gums?
  - What do you do regularly to look after your teeth and gums?
    - - At home? Cleaning/flossing/diet etc. how/when/how often
      - Seeing dentist/hygienist? how/when/how often
  - Has any of this changed since you were told of your risk of RA?
  - Does anything get in the way of you looking after your teeth and gums and/or visiting dentist?
    - - Health issues
      - Dexterity in hands
      - Other commitments (work/family) and time
      - Are you a regular attender at the dentist?
      - Do you have a dentist? Can you get to the dentist?
      - Is cost an issue at the dentist or for making any of the suggested changes to the way you look after your teeth and gums?

**Acceptability of periodontal care in treatment plan of individuals at risk of developing RA**

- Knowing that there is a link between your gums and developing RA do you think you should be offered help with this?
  - - How would you want that help? What’s the main challenge for you?
    - At home – remembering/cleaning/flossing
    - Better toothbrush or other things to use at home
    - Getting a dentist? Getting to a dental appointment?
    - Cost of dentist? Cost of getting to a dentist?
    - Advice from rheumatology team?
    - Further information? From who? And how want it – verbal/written/app?

**Shared risk factors**

Have you heard about any other links with developing RA?

- Are you aware of the effect of smoking on your health in particular your mouth
  - What about the link with developing RA?
  - What do you think about help with stopping smoking being offered as part of the study?

• Are you aware of the effect that your diet and gut bacteria has on your health?

- What about the link that your diet and gut bacteria has to developing RA?
- Would you also be willing to change your diet following advice from a health professional, take a prescribed supplement (purified yoghurt) or an antibiotic to reduce your risk?

**Factors influencing your participation in the research project**

**Knowledge of and weighing up pros and cons of preventative intervention**

- - What encourages/would encourage you to participate in a study that would provide dental and in particular gum treatment with a view of potentially helping your joints (Caveat)
    - Pain free treatment
    - Resolving your pain
    - On-off appointment vs longer-term treatment
    - Would gender of the dentist influence your decision
  - What would be the main challenge for you to taking part in such a study?
    - Anxiety – Of dentist? Treatment? Pain? Discomfort?
      - Types/cause/experiences
      - Specific fears
      - Onset
      - Coping Strategies
    - Barriers to care if any
    - Language/cultural difficulties
    - Fear/embarrassment
    - Time commitment

What could we do to overcome these challenges and encourage you to take part?
